# Apport des moyens endoscopiques dans la dilatation des sténoses caustiques de l’œsophage

**DOI:** 10.11604/pamj.2016.23.24.8506

**Published:** 2016-02-03

**Authors:** Togo Seydou, Ouattara Moussa Abdoulaye, Li xing, Sanogo Zimogo Zi, Koumaré sekou, Yang Shang Wen, Sankare Ibrahim, Toure Cheik Ahmed Sekou, Maiga Ibrahim Boubacar, Jacque Saye, Dakouo Dodino Jerome, Toure Ousmane Dantoumé, Yena Sadio

**Affiliations:** 1 Service de Chirurgie Thoracique, Hôpital du Mali, Bamako, Mali; 2 23^ème^ Mission Médicale Chinoise, Hôpital du Mali, Bamako, Mali; 3 Service de Chirurgie « A » CHU du Point G, Bamako, Mali; 4 Service de Santé Publique et de Statistique Hôpital du Mali, Bamako, Mali

**Keywords:** Œsophage, sténoses caustiques, dilatation, endoscopiques, Esophagus, caustic stenosis, dilatation, endoscopic

## Abstract

**Introduction:**

Toutes les sténoses symptomatiques de l’œsophage peuvent être dilatées par voie endoscopique. Nous évaluons l'apport des moyens endoscopiques dans la prise en charge de la dilatation œsophagienne pour sténose caustique de l’œsophage (SCO) au Mali.

**Méthodes:**

IL s'agissait d'une étude descriptive et prospective réalisée dans le service de chirurgie thoracique à l'hôpital du Mali. Au total 46 dossiers cliniques de patients on été enregistrés et subdivisés en 4 groupes en fonction de la topographie des lésions cicatricielles. Le nombre de cas d'assistance endoscopique réalisé a été déterminé afin de comprendre l'apport des moyens endoscopiques dans le succès de la dilatation des SCO. Pour les 2 différentes méthodes de dilatation utilisées, le résultat du traitement et le coût ont comparés.

**Résultats:**

La FOGD a été utilisée dans 19 cas (41.30%) de dilatation avec la bougie de Savary Guillard et dans 47.82% des cas dans la dilatation de Lerut. La vidéo-laryngoscopie a été utilisé 58.69% des cas de dilatation à la bougie de Lerut. Le passage de guide métallique et / ou de fil-guide a été réalisée dans 39.13% avec la vidéo laryngoscopie et dans 58.68% avec la FOGD. Dans la comparaison des deux méthodes, il existe une différence significative dans la survenue des complications (p=0.04075), l'anesthésie générale (p=0.02287), l'accessibilité à la méthode (p=0.04805) et la mortalité (p=0.00402).

**Conclusion:**

La SCO est une pathologie grave et sous évaluée au Mali. Les moyens endoscopiques contribuent considérablement au succès de la dilatation œsophagienne pour sténose caustique dans les différentes méthodes utilisées.

## Abstract (en)

**Introduction:**

The aim of this work was to present the contribution of the endoscopy in the management of esophageal dilatation for caustic esophageal stenosis (CES).

**Methods:**

This was a descriptive and prospective study in the thoracic surgery department at the Hospital of Mali. A total of 46 cases of CES is recorded and divided into 4 groups according to the topography of the esophageal lesions. For the different methods of dilatation the number of performed endoscopic support was determined to understand the contribution of endoscopic means in the success of dilatation for CES. The outcome, complications and mortality in the two methods were compared.

**Results:**

Fibroscopy was used in 41.30% of patients with Savary Guillard dilators and in 47.82% of patients with Lerut dilators. Video laryngoscopy was used in 58.69% of patients who underwent dilatation with Lerut dilators. The passage of the guide wire was performed in 39.13% under video laryngoscopy and 58.68% under fibroscopy. In comparison of the two methods, there is a significant difference in the occurrence of complications (p=0.04075), general anesthesia (p=0.02287), accessibility (p=0.04805) and mortality (p=0.00402).

**Conclusion:**

The CES is a serious disease and under evaluated in Mali. The endoscopies contribute significantly to the success of esophageal dilatation for caustic stenosis in the different methods we used.

## Introduction

L'objectif de la dilatation dans la SCO est d'obtenir la disparition ou l'amélioration de la dysphagie et dans les sténoses d'origine inconnu permettre le passage de l'endoscope. Toutes les sténoses symptomatiques peuvent être dilatées par voie endoscopique et peut être proposé en première intention du faite de sa bonne tolérance et de la simplicité de sa mise en œuvre. [1]. Les indications thérapeutiques dans la SCO restent source de polémique dans la littérature [1, 2]. En effet, plusieurs types de traitement pour la SCO existent mais l'approche thérapeutique conservatrice, par dilatation est celle que nous avons privilégiée à cause de l'inaccessibilité en milieu africain souvent très paupérisé de certaines techniques trop coûteuses. Cependant l'apport des moyens endoscopiques pour mener à bien cette dilatation n'est pas négligeable et n'est pas trop souvent mis en exergue dans la littérature. Le but de ce travail est de présenter l'apport des moyens endoscopiques dans l'efficacité de la dilatation des SCO dans notre contexte.

## Méthodes

De janvier 2011 à janvier 2015, nous avons colligés 46 dossiers cliniques de patients et pris en charge. Il s'agissait d'une étude descriptive et prospective réalisée dans le service de chirurgie thoracique à l'hôpital du Mali. Les patients ont été subdivisés en 4 groupes en fonction de la topographie des lésions cicatricielles pour les besoins de l’étude: groupe 1 (sténose caustique limitée à l′œsophage; n= 34); groupe 2 (SCO avec lésions ORL; n= 8); groupe 3 (SCO avec lésions gastriques; n= 3) et groupe 4 (SCO avec lésions ORL et gastriques; n= 1). Tous les patients ont été reçus au stade de sténose caustique cicatricielle de l’œsophage avec ou sans gastrostomie d'alimentation. Un bilan biologique standard et une consultation anesthésique ont précédé la prise en charge. Une fibroscopie œso-gastro duodénale(FOGD) et/ou un transit oeso-gastroduodénal (TOGD) a été réalisé selon la demande du médecin. Une préparation nutritionnelle (orale, entérale ou parentérale) a été effectuée. La gastrostomie d'alimentation a été réalisée mais pas systématique et considérée comme définitive en cas d'impossibilité de dilatation. La première méthode de dilatation œsophagienne a été celle réalisée avec la bougie de Tony Lerut qui est celle de Savary Guillard modifiée par le Pr Tony Lerut, spécialiste des pathologies de l’œsophage de la Belgique. A l'aide d'un guide métallique, un fil-guide est placé passant par la bouche sortant par l'orifice de la gastrostomie qui sera lié à un bout de la bougie et servir pour sa traction pendant la dilatation. Cette méthode de dilatation a été réalisée le plus souvent avec l'assistance des moyens endoscopiques (FOGD et vidéo-laryngoscopie).

**Figure 1:**
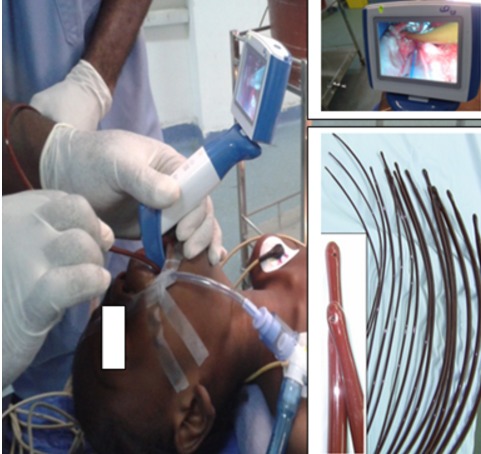
Dilatation à la bougie de Lerut par assistance vidéo laryngocopique

**Figure 2:**
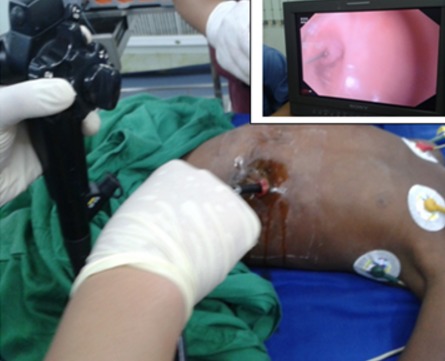
Fibroscopie rétrograde avec passage de guide

La deuxième méthode a été la dilatation avec la bougie de Savary Guillard qui a été exclusivement réalisée à l'aide de la FOGD. Elle a été réalisée initialement pour mettre en place le fil-guide au bloc opératoire pour les sténoses serrées afin de pouvoir réaliser les séances futures de dilatation à la bougie de Lerut. Lorsque l'indication le permettait, elle était utilisée pour dilater les patients dont les sténoses étaient moins serrées et qui pouvaient supporter la dilatation sans anesthésie générale. La vidéo laryngoscopie a été utilisée pour assistance à la dilatation avec la bougie de Lerut au cours des séances itératives (Figure 1). La FOGD a été fréquemment utilisée par voie antérograde ou rétrograde lorsque la sténose était infranchissable. La voie antérograde était la voie conventionnelle passant par la bouche. La voie rétrograde a consisté au passage du fibroscope flexible par l'orifice de la gastrostomie afin de passer le guide de l'orifice de la gastrostomie à la bouche à et permettre la mise en place du Fil-guide pour la traction de la bougie de Lerut (Figure 2). La vidéo-laryngoscopie et la FOGD étaient souvent couplées. La gastro-entéro-anastomose (GEA) a été réalisée en cas de sténose gastrique ou bulbaire associée si une alimentation orale était possible après la dilatation de l’œsophage. Le résultat du traitement a été considéré comme réussi lorsque les patients étaient capables de maintenir une alimentation orale solide ou semi-solide sans autres traitements. Les données cliniques, les caractéristiques de la sténose œsophagienne (siège, nombre), les lésions caustiques extra œsophagiennes associées (ORL, gastriques), les données du traitement de la sténose, les complications post thérapeutiques, le résultat fonctionnel (alimentation, regain pondérale) et le coût direct de la prise en charge ont été analysés. La mortalité et la morbidité ont été rapportées à chaque groupe et aux différentes méthodes de dilatation. Pour les différentes procédures de dilatation le nombre d'assistance endoscopique réalisé a été déterminé afin de comprendre l'apport des moyens endoscopiques dans le succès thérapeutique des différentes méthodes de dilatation. Les résultats des 2 méthodes de dilations utilisées ont été également comparés. Les variables ont été analysées en utilisant le test exact de Fisher. Toutes les opérations statistiques ont été réalisées avec le logiciel open EPI. La valeur de P < 0,05 a été considérée comme significative pour toutes les procédures.

## Résultats

### Caractéristiques de la population

Durant la période d’étude, 46 patients âgés de 06 mois à 21 ans (moyenne: 6 +/- 4 ans) ont été traités par l’équipe de chirurgie thoracique pour sténose caustique de l’œsophage. Il y avait 33 garçons et 13 filles. Le sexe ratio était de 2.54 en faveur du sexe masculin. Le délai d’évolution moyenne de la brûlure caustique a été de 6 mois +/-2 (extrêmes: 4 mois et 8 mois). La dénutrition était constante chez l'ensemble des patients et 18 patients (39.13%) sont arrivés en consultation dans le service avec une gastrostomie d'alimentions. Les patients qui avaient bénéficiés d'un traitement traditionnel (décoctions, huile de palme, kaolin, eau de mer, etc…) avant de se faire consulter dans un centre médicalisé représentaient 92%. Chez une adolescente l'ingestion de produit caustique était volontaire à but suicidaire pour refus à un mariage forcé.

### Résultats technique et clinique de la dilatation

La sténose œsophagienne était isolée dans 34 cas (73.91), associée à des lésions ORL dans 16 cas (34.78%) et gastriques dans 3 cas (6.52%). La dysphagie était totale dans 32.60% des cas et la sténose était étagée chez 15 patients (32.60%) (Tableau 1). La FOGD a été utilisée dans l'ensemble des cas (19 patients) de dilatation avec la bougie de Savary Guillard et dans 47.82% des cas (22 patients) dans la dilatation de Lerut. La vidéo-laryngoscopie a été utilisé dans l'ensemble des cas (27 patients) de dilatation à la bougie de Lerut et seulement dans un cas avec la bougie de Savary Guillard. Dans les deux moyens endoscopiques utilisés, le passage de guide métallique et / ou de fil-guide a été réalisée dans 39.13% avec la vidéo laryngoscopie et dans 58.68% avec la FOGD. La survenue de complication et de décès pour les 2 moyens endoscopiques et les 2 méthodes de dilatation est consignée dans le Tableau 2. Dans la comparaison des deux méthodes, il existe une différence significative dans la survenue des complications (p=0.04075), l'anesthésie générale (p=0.02287), l'utilisation de la vidéo-laryngoscopie (p < 0.0001) l'accessibilité à la méthode (p=0.04805), la mortalité (p=0.00402) et du cout moyen (p=0.02960). Par contre il n'ya pas de différence dans le regain pondérale, la reprise alimentaire orale et les différents moyens endoscopiques utilisés (Tableau 3). La reprise alimentaire orale a été effective chez 39 patients (84.78%).

**Tableau 1:** Caractéristiques anatomo-cliniques des lésions caustiques

Caractéristiques	Effectif (%)
**Type de dysphagie**	
Totale	15(32.60)
Aux aliments semi-solides	13(28.26)
Aux aliments solides	18(39.13)
**Topographie des lésions œsophagiennes**	
Cervicale	14 (30.43)
Thoracique	15 (32.60)
Cardia	2 (4.34)
Étagée	15 (32.60)
**Classification des lésions caustiques**	
Groupe 1(G1)=SCO isolée	34(73.91)
Groupe 2(G2)=SCO + lésions ORL	8(17.39)
Groupe 3(G3)=SCO + lésions Gastriques	3(8.69)
Groupe 4(G4)=SCO + lésions ORL et gastriques	1(2.17)

SCO* : sténose caustique de l’œsophage

**Tableau 2:** Résultats des différents gestes réalisés en fonction des groupes

Gestes de dilatation	G1	G2	G3	G4	Effectif (%)	Compl* (%)	Décès (%)
*Bougie Tony Lerut Dilatation seule	4	-	-	-	4 (8.69)	-	-
Dilatation reproductible + gastrostomie	14	-	-	-	14(30.43)	4(8.69)	2(4.34)
Dilatation ( tentative) + gastrostomie définitive	-	5	-	-	5(10.86)	5(10.86)	4(8.64)
Dilatation + GEA	1	-	-	1	2(4.34)	-	-
Dilatation + gastrostomie + GEA	-	-	1	-	1(2.17)	-	-
Dilatation + jejunostomie	-	-	1	-	1(2.17)	1(2.17)	1(2.17)
*Bougie Savary Guillard Dilatation seule	15	-	1	-	16(34.78)	1(2.17)	1(2.17)
Dilatation + Gastrostomie	-	3	-	-	3(6.52)	2(4.34)	-
**Gestes endoscopiques**							
*Fibroscopie œsogastroduodénale Aspiration débris alimentaire	8	-	-	-	8(17.39)	-	-
Passage de guide métallique + fil-guide	11	3	-	-	14(30.43)	2(4.34)	-
Incision de cicatrices orificielles + fil-guide	4	2	1	1	8(17.39)	2(4.34)	1(2.17)
Contrôle hémorragique	2	2	1	-	5(10.86)	-	-
Recherche compl* de fistule œsophagienne	1	-	-	-	1(2.17)	-	-
Dilatation Savary pour passage fil-guide - Lerut	5	-	-	-	5(10.86)	1(2.17)	-
Dilatation bougie de Savary G.	15	3	1	-	19(41.30)	1(2.17)	1(2.17)
*Vidéo-laryngoscopie Passage de fil-guide + dilatation bougie Lerut	15	3	-	-	18(39.13)	4(8.69)	2(4.34)
Dilatation directe à la bougie de Lerut	5	2	1	-	8(17.39)	1(2.17)	1(2.17)
Passage de guide + dilatation bougie Savary G	1	-	-	-	1(2.17)	0(0)	0(0)

* Compl : complications post thérapeutiques GEA : gastro-antéro-anastomose G1: Groupe 1

**Tableau 3:** Données comparées de la dilatation (Dilatation bougie Savary versus bougie Tony Lerut)

Données	Dilatation Bougie Savary Guillard n= 19	Dilatation Bougie Tony Lerut, n=27	*P*
*Dilatation	19(41.30)	27 (58.69)	0.2043
*Complications	3 (6.52)	10 (21.73)	**0.04075**
-fistules œsophagienne	1 (2.17)	2 (4.34)	0.20080
-infection pulmonaire (inhalation)	0	1 (2.17)	0.33333
- dénutrition sévère	2(4.34)	5 (10.86)	0.08981
- hémorragies	0	1 (2.17)	0.33333
- septicémie	0	1 (2.17)	0.33333
*Reprise alimentaire orale	18(39.13)	20 (43.47)	0.06976
*Regain pondérale	18(39.13)	20 (43.47)	0.06976
*Anesthésie générale	11(23.91)	27 (58.69)	**0.03287**
*FOGD	19 (41.30)	22 (47.82)	0.31803
*Vidéo-laryngoscopie	1(2.17)	27(58.69)	**P < 0.0001**
*Faisabilité	18 (39.13)	20 (43.47)	0.16976
*Accessibilité	19 (41.30)	27(58.69)	**0.04805**
*Reproductibilité	18 (39.13)	20(43.47)	0.40201
*Mortalité	1 (2.17)	7 (15.21)	**0.01402**
*Coût moyen (Euro)	34.38 ± 4	139.13 ±15	**0.02960**

FOGD : Fibroscopie oesogastroduodenale

### Suivi des patients

L’évolution était souvent favorable après dilatation dans les 2 méthodes utilisées. Il y a eu 4 cas de resténose (8.69%) qui ont été secondairement redilatés. Les complications représentaient 28.25%. Le délai moyen de suivi des patients étaient de 11 mois +/- 4. La mortalité était de 17.38% dont 15.21% de décès survenus dans la dilatation à la bougie de Lerut et 2.17% à la bougie de Savary Guillard. La mortalité a été plus importante chez les patients qui avaient une gastrostomie définitive.

## Discussion

Les sténoses œsophagiennes de l'enfant d'origine caustique sont parmi les plus fréquentes en Afrique et dans les pays en voie de développement [3]. Sa survenue est presque toujours accidentelle mais il faut cependant ne pas oublier les tentatives d'infanticide ou de suicide chez l'enfant ou chez l'adulte, avec le degré de lésions lié à la nature et la quantité de caustique ingérée [4]. Certaines réalités socio-économiques africaines et en particulier la région subsaharienne, peuvent favoriser la survenue d'un accident domestique caustique chez l'enfant. Ce sont entre autres: le faible niveau d’éducation, les conflits, la polygamie, la famille nombreuse, le taux de fécondité élevé, l'errance des enfants, la criminalité croissante ou l'existence d'une entreprise familiale génératrice de revenus utilisant les produits corrosifs à domicile telle que la teinture, la fabrication du savon ou de soude caustique à usage alimentaire (préparation d'aliment locale à base de mil appelé le Tô). Pour certains auteurs, le mauvais itinéraire dans la prise en charge médicale en est un facteur, de même que le recours fréquent des patients à la médecine traditionnelle sont des raisons qui favorisent les complications [3, 5].

Dans notre travail, les patients arrivent constamment en chirurgie à un stade tardif après l'installation des conséquences de la dysphagie dans plus de la moitié des cas. Ces facteurs grèvent négativement le pronostic et explique la morbidité et la mortalité élevées dans nos régions. La coexistence de la SCO avec les lésions ORL et/ou gastriques est une situation préoccupante. Elle est reconnue comme étant un facteur d'aggravation et de co-morbidité [6, 7]. Dans la littérature, plusieurs modalités de dilatations utilisant différents types de dilatateurs ont été proposées [4, 5, 8]. Mais dans notre travail deux type de dilatation ont été utilisés mais l'utilisation de la bougie de Savary-Gilliard modifiée de Lerut guidée par du fil sans fin associé ou non à une gastrostomie a montré plusieurs avantages dans un milieu peu équipé. Elle a été la plus utilisée parcequ'elle est simple de pratique, facile à reproduire et bien tolérée. Elle permet une reprise alimentaire le même jour [8]. Les mérites de la dilatation ont été signalées en Afrique depuis 1972 par [9]. La dilatation œsophagienne a un avenir très prometteur dans notre contexte puisque sa technique continue d’être améliorée par le perfectionnement de l'endoscopie interventionnelle qui permet actuellement de re perméabiliser dans de bonne conditions les SCO longues, complexes et étagées par la mise en place de prothèses ou par dilatation [10]. Les progrès actuels de la fibroscopie souple et la modernisation des dilatateurs de l’œsophage permettent une dilatation instrumentale avec un maximum d'efficacité et un minimum de complications.

La correction de la dénutrition est faite par une alimentation entérale grâce à la réalisation de la gastrostomie (73.7% des cas) comme dans l’étude de Contoni [3]. En plus de son exécution simple, la gastrostomie permet d'alimenter les patients par les aliments locaux (en liquide ou liquéfiés) et de faire passer le fil-guide en cas de dilatation par voie antérograde ou rétrograde. En effet plus de complications iatrogènes sont observées parce que le passage de fil-guide ou guide simple qui est en fait le pilier de la réussite de la dilatation dans les 2 méthodes que nous avons utilisées est souvent très complexe avec beaucoup de manœuvres. La survenue de complications est plus importante dans la dilatation avec la bougie de Lerut surtout avec la possibilité de nombreuses manœuvres car tous les patients sont sous anesthésie générale. La comparaison des complications (p=0.04075) et de l'anesthésie générale (p=0.02287) dans les 2 méthodes est significatif. La comparaison de la mortalité dans les deux méthodes est statistiquement significatif (p=0.01402). La fibroscopie œsophagienne et la vidéo-laryngoscopie ont donc beaucoup été utilisées à cause de la vision indirecte qu'elles offrent lors des différentes manœuvres. La première séance de dilatation nécessite le plus souvent le recours à ces moyens endoscopiques d'autant plus que le passage de guide métallique ou du fil-guide reste difficile et nécessite certains gestes préalables sous endoscopie. Le plus souvent les débris alimentaires peuvent fermer la lumière de l’œsophage sténosée et seule la fibroscopie œsophagienne permet d'aspirer les débris alimentaires et salivaires afin de rendre perméable l’œsophage et permettre le passage du fil-guide. La FOGD est indispensable à une bonne dilatation par la bougie de Savary Guillard. Avec la bougie de Lerut, la FOGD est fréquemment utilisée pour la dilatation initiale mais elle devient peu fréquente pour les séances répétitives de dilatation dès que le fil-guide est mis en place. Dans la comparaison des deux méthodes de dilatation que nous avons utilisées, l'utilisation de la FOGD est presque similaire. La fibrose sténosante de la lumière de l’œsophage est souvent infranchissable et seul la FOGD permet d'obtenir des moyens de dilatation ou de passage du fil-guide [9]. Dans notre étude la FOGD a servi entre autre à inciser les fibroses cicatricielles, à évaluer le diamètre des orifices de sténose afin de pouvoir faire le choix du calibre du guide, à faire l'hémostase lors des hémorragies pendant la dilatation et souvent de poser le diagnostique des complications de la dilatation telles que les perforations. Dans la dilatation de l’œsophage à la bougie de Lerut La FOGD réalisés de façon antérograde comme rétrograde ont pour seule objectif de pouvoir faire passer le fil-guide. Le passage du fil-guide par voie rétrograde n'a été possible que seulement sous FOGD chez tous nos patients qui ont bénéficié de cette technique.

Dans notre travail la dilatation à la bougie de Savary Guillard par l'aide de la FOGD a occupée une place importante. Cette méthode de dilatation est possible exclusivement que par l'aide de la FOGD [4, 8, 9]. Elle a substitué le plus souvent à la dilatation de Lerut avec un cout plus accessible. Elle a permis de réaliser une dilatation initiale au bloc opératoire sous anesthésie générale pour pouvoir faire passer le fil -guide ou de réaliser des dilatations dans la salle d'endoscopie avec absence d'anesthésie générale surtout chez les adolescents (7 à 15 ans) et les adultes. Il est aisément compréhensible que l'endoscopie œsogastroduodénale garde une place majeure dans la réussite de la dilatation des SCO comme le confirme l’étude de Hamza et Al. [4] La méthode est facilement reproductible mais l'accessibilité reste encore difficile à cause de la pauvreté de ressource humaine qualifiée dans les dilatations endoscopiques dans le milieu africain. En plus, les quelques rares endoscopistes sont souvent trop sollicité. La vidéo laryngoscopie a été un véritable outil utilisé dans la dilatation à la bougie de Lerut. Elle a été utilisée dans toutes les séances de dilation possible à la bougie de Lerut avec ou sans gastrostomie. Elle a permis d'objectiver de façon indirecte le passage de la bougie au niveau de la bouche de Killian (chose qui n'est pas toujours aisée lorsqu'il existe des lésions ORL associées) et aussi d'aspirer les secrétions salivaires qui masquent l'orifice. Son utilisation comparatif dans les deux méthodes de dilatation est statistiquement significatif (P < 0.0001). En définitif, la stratégie de la prise en charge d'une SCO doit se concevoir dans un contexte de concertation pluridisciplinaire incluant au moins le pédiatre, l'endoscopiste digestif, le radiologue, l'anesthésiste, le chirurgien avec la participation active des familles [7]. Cette attitude participative a le mérite de rendre accessible le traitement de la SCO à un maximum de victimes à moindre coût.

## Conclusion

La sténose caustique est une pathologie grave et sous évaluée au Mali. Sa morbi-mortalité reste élevée et sa gravité est en rapport avec la prise en charge tardive et la présence de lésions caustiques associées (ORL et gastrique). La dilatation œsophagienne est un moyen thérapeutique simple, efficace et applicable dans notre contexte d'exercice. Cependant l'apport des moyens endoscopiques dans le succès de la dilatation œsophagienne pour sténose caustique quelles que soit les différentes méthodes utilisées est considérable. Toutefois un accent particulier doit être mis sur la conduite d'un programme de prévention à échelle.

### Etat des connaissance sur le sujet

- Les sténoses caustiques de l’œsophage (SCO), constituent un problème grave de santé publique en Afrique
- La morbi-mortalité reste élevée dans la prise en charge chirurgicale
- La prise en charge reste difficile surtout dans les pays en voie de développement

### Contribution de notre étude à la connaissance

- Les méthodes de dilatation des SCO sont applicables dans notre contexte avec de bons résultats et le résultat de la dilatation est meilleur sous assistance endoscopique
- L'endoscopie contribue au succès de la dilatation quel que soit les différentes méthodes de dilatation utilisées
- La morbi-mortalité est faible avec la dilatation à la bougie de Savary Guillard comparée à celle de Lerut
